# Receptor and Channel Heteromers as Pain Targets

**DOI:** 10.3390/ph5030249

**Published:** 2012-02-23

**Authors:** Kelly A. Berg, Amol M. Patwardhan, Armen N. Akopian

**Affiliations:** 1 Department of Pharmacology, University of Texas Health Science Center at San Antonio, San Antonio, TX 78229, USA; Email: berg@uthscsa.edu (K.A.B.); 2 Department of Anesthesiology, Arizona Health Sciences Center, Tucson, AZ 85724, USA; Email: amol.arizona@gmail.com (A.M.P.); 3 Department of Endodontics, University of Texas Health Science Center at San Antonio, San Antonio, TX 78229, USA

**Keywords:** pain, receptors, channels, sensory neurons, heteromers

## Abstract

Recent discoveries indicate that many G-protein coupled receptors (GPCRs) and channels involved in pain modulation are able to form receptor heteromers. Receptor and channel heteromers often display distinct signaling characteristics, pharmacological properties and physiological function in comparison to monomer/homomer receptor or ion channel counterparts. It may be possible to capitalize on such unique properties to augment therapeutic efficacy while minimizing side effects. For example, drugs specifically targeting heteromers may have greater tissue specificity and analgesic efficacy. This review will focus on current progress in our understanding of roles of heteromeric GPCRs and channels in pain pathways as well as strategies for controlling pain pathways via targeting heteromeric receptors and channels. This approach may be instrumental in the discovery of novel classes of drugs and expand our repertoire of targets for pain pharmacotherapy.

## 1. Introduction: Receptor and Channel Heteromers as Therapeutic Targets for Pain

Receptors and channels are traditional targets for drug development in the battle against a variety of diseases. Current strategies for drug development assume that receptors and channels are monomers or homomers. Conventional models propose that monomeric G-protein coupled receptors (GPCRs) control the sequential activation of cellular signaling cascades and eventually channel-effectors. Similarly, channels acting as monomers (or homomers) possess defined pharmacological, biophysical and functional properties and are regulated in cell-specific ways. Evidence accumulated in recent years suggests that these models are too simplistic to explain the functional flexibility of these receptors and channels. The current view of receptor and channel organization suggests that a majority of receptors and especially channels are present as dimers or heteromers in cells [1,2,3,4,5,6]. Receptor and channel heteromer complexes are formed by direct interaction with either members of the same receptor/channel family or members of structurally and functionally differing families [7,8,9,10,11]. In certain cases, heteromerization is a requirement for the formation of functional receptors/channels. Thus, the NR1 subunit of NMDA receptors does not form glycine-glutamate-responsive channels as it requires the presence of a NR2 subunit to do so [12]. However, a majority of receptors and channels form non-obligatory heteromers which display unique and specific pharmacological, biophysical, regulatory and functional characteristics. Therefore, heteromer complexes may be considered to be novel and distinct entities with the potential for playing unique physiological roles during normal and pathological conditions.

The aim of this review is to present advantages as well as strategies for controlling pain pathways via targeting heteromeric receptors and channels. This approach may be instrumental in the discovery of novel drugs for pain therapy. Although the occurrence of receptor and channel heteromerization is now well recognized (see reviews [1,4,7,8,13]), we are just now beginning to understand the characteristics of different receptor and channel heteromerization. Thus, the proportion of receptor/channel monomers (or homomers) *versus* heteromers in the plasma membrane of actual physiological cells is still unknown for the majority of receptors and channels. Furthermore, there are functional, regulatory and pharmacological outcomes of heteromerization that are beginning to be acknowledged for a set of receptors and channels. Nevertheless, this area of research is well worth the effort and investment, as targeting receptor/channel heteromers may provide a completely new therapeutic strategy for treatment of different pain conditions.

There are several reasons for engaging in drug discovery which targets receptor and channel heteromer. First, localization of heteromers (*i.e*., tissue and cell-specificity) may be restricted to fewer cell types than the expression of monomeric and homomeric subunits that comprise the heteromers. More specific expression patterns may provide for a reduction of side-effects in response to heteromeric selective drugs. For example, highly selective TRPV1 receptor antagonists were developed with the intent to block inflammation-induced thermal and mechanical hyperalgesia [14,15,16]. Unfortunately, the TRPV1 receptor is also involved in the control of body temperature [17] and several critical functions of the CNS [18,19,20]. Thus, TRPV1 receptor antagonists produced unwanted side effects such as fever [21]. Drugs selective for TRPV1 heteromers [22,23] may potentially avoid this side-effect, as TRPV1-heteromers could express selectively in nociceptors and/or skin, but not cells engaged in other body functions [4]. Second, there is increasing evidence that a receptor or channel heteromer is an entity with a distinct pharmacology and/or functionality [1,5,10,24,25]. Heteromers may modulate a given physiological process in a substantially different way compared to monomer/homomer subunits composing these heteromers. As a consequence to unique physiological effects, heteromer-selective drugs may not produce side effects attributed to monomeric or homomeric receptors/channels. Third, the physiological role of a heteromer may include modulation of receptor homomer function [9,26]. For example, heteromerization between TRPV1 and TRPA1 channels can substantially affect the calcium signaling pathways of TRPA1 homomers [26,27]. Thus, it is possible that the signaling pathway activated by the heteromer may be of high importance in some physiological processes, but not others, which are governed by homomers. For example, the D1-D2 dopamine receptor heteromer plays a role in LTP, while the D2–D5 heteromer’s role is restricted to control of motor activity [26]. This underscores the potential for new therapeutic strategies specifically targeting one signaling pathway among the variety of pathways that homomer receptors/channels and their heteromers offer. Altogether, receptor/channel heteromer-selective drugs offer more flexible control of receptor/channel functions, with the potential for a lower incidence of side effects.

## 2. G-Protein Coupled Receptor Heteromers as Therapeutic Targets for Pain

G protein coupled receptors (GPCRs) are the largest family of cell surface receptors and are the most common targets for therapeutic drugs including drugs used for treatment of pain [28]. Receptor activation in response to ligand binding as well as constitutive receptor activity (*i.e*., ligand-independent receptor activity) leads to coupling and activation of heterotrimeric G proteins which in turn regulate numerous intracellular signaling events. G proteins are grouped into four subfamilies: G_i/o_, G_s_, G_q/11_ and G_12/13_. Most analgesics that target GPCRs activate G_i/o_-coupled receptors including opioid, cannabinoid, alpha2-adrenergic, somatostatin, muscarinic acetylcholine, gamma-aminobutyric acid B (GABA_B_), and groups II and III metabotropic glutamate receptors [29]. It is important to note that GPCRs can also couple to other signal transduction molecules in addition to G proteins.

Originally thought to function only as receptor monomers, it is now known that GPCRs also function as homo- and heter-odimeric complexes (between different receptor subtypes) and perhaps even as higher order oligomers [6]. Here, the term receptor homomer or heteromer will be used to refer to dimeric or higher order complexes. Receptor heteromers frequently display unique signaling characteristics and pharmacological properties that differ from those of the individual protomers and thus can be viewed as distinct receptor entities [30,31,32,33,34,35]. It has been suggested that these unique or “signature” signaling and pharmacological characteristics can be used to determine receptor heteromer involvement in a given physiological function such as pain modulation [5]. Further, an important feature of receptor heteromers from a pharmacological standpoint is that allosteric interactions can occur between the individual protomers. As a result, the presence of one protomer can alter the affinity and/or efficacy of ligands that bind to the other protomer. Moreover, conformational changes produced by ligand occupancy of one protomer can alter ligand affinity/efficacy at the second protomer [6,36,37,38]. Such allosteric interactions between protomers may contribute to the distinct signaling characteristics of receptor heteromers. Although there is evidence for heteromerization for all of the receptors mentioned above, given the significance of opioid receptors in pain modulation, here we will focus on heteromers that include opioid receptors.

### 2.1. Opioid Receptor Heteromers

All three major opioid receptors are involved in modulation of various pain states and are expressed in brain, spinal cord as well as dorsal root (DRG) and trigeminal ganglion (TG) sensory neurons [39,40]. There are several lines of evidence that support the existence of opioid receptor heromers. First, a pre-requisite for heteromer formation is cellular co-localization and all three opioid receptor subtypes have been found to co-express (to varying extents) in spinal cord and in peripheral sensory neurons [41,42,43]. Second, pharmacological characteristics of opioid ligands tested *in vivo* have not always matched those obtained with individual opioid receptors expressed in heterologous expression systems [44,45]. Many of these pharmacological differences can be accounted for by receptor heteromerization [46]. Third, biochemical studies both *in vitro* and *in vivo* have shown that MOR, DOR and KOR form receptor heteromers with each other as well as with other GPCR targets for analgesic drugs, including cannabinoid and alpha adrenergic receptors.

### 2.2. MOR-DOR Receptor Heteromers

Many early pharmacological studies have shown that co-administration of DOR agonists as well as antagonists increases analgesic efficacy of morphine while decreasing unwanted side effects including tolerance and the dependence liability (for a review see [45]). Subsequently, studies done with DOR null-mutant (KO) mice and experiments using antisense oligonucleotides to reduce DOR expression have shown that the presence of DOR alters the analgesic efficacy of morphine [47,48]. Similarly, DOR-mediated antihyperalgesia appears to require the presence of MOR, because in MOR KO mice the analgesic effects of DOR agonists were lost [49]. Such *in vivo* studies have provided evidence for interactions between MOR and DOR that appear to be important for the efficacy of opioid analgesics.

Heteromer receptor formation between MOR and DOR has been demonstrated directly *in vitro* with transfected COS-7 cells [50] and HEK 293 cells [51]. Although there has been some controversy as to whether MOR and DOR receptors co-localize in DRG neurons and spinal cord [52], this debate has largely been put to rest by reports of Devi and coworkers [53] and Hokfelt and coworkers [43] showing that the opioid receptors do indeed co-localize to nociceptive sensory neurons in the DRG and spinal cord. Furthermore, heteromer formation between MOR and DOR has been demonstrated directly in spinal cord neurons [54], DRG sensory neurons [53] as well as rostral ventral medulla (RVM) neurons [53], a key relay nucleus for pain perception.

In heterologous expression systems, novel pharmacology and G protein coupling is observed for the MOR-DOR heteromer that is distinct from that of activation of either MOR or DOR alone. Agonist, but not antagonist, binding affinity to MOR is decreased in the presence of DOR co-expression [50,55] Occupancy of DOR with either an agonist (deltorphin II), antagonist (TIPPψ) or inverse agonist (ICI174864) enhanced the maximal binding of [^3^H]-DAMGO (selective MOR ligand) with little or no change in [^3^H]-DAMGO binding affinity [51,54]. In addition, the maximal binding of the selective DOR agonist, [^3^H]-deltorphin II, was increased in the presence of the selective MOR antagonist, CTOP [51] indicating that changes in ligand binding are reciprocal between the protomers of the MOR-DOR heteromer. In a recent study, Gomes *et al*. [56] demonstrated that the dissociation kinetics of MOR and DOR radioligands were altered by ligands for the opposite protomer. Changes in dissociation kinetics are a hallmark of allosterism [57,58] and strongly suggest that reciprocal allosteric interactions occur between the MOR and DOR protomers of the MOR-DOR heteromer which may contribute to the unique MOR and DOR ligand pharmacology of the receptor heteromer [57,58].

Distinct signaling characteristics of MOR-DOR heteromer receptor activation may also be evident *in vivo*. Thus, DOR antagonists have been shown to enhance MOR agonist-mediated GTPγs binding in spinal cord membranes. ß-arrestins were originally identified as molecules involved in desensitization of GPCRs following prolonged or repeated activation [59], which leads to drug tolerance. In contrast to the individual receptors, MOR-DOR heteromerization in CHO cells recruits β-arrestin-2 constitutively, leading to differences in monomer/homomer *versus* receptor heteromer-mediated ERK activation *in vitro* [60]. Moreover, ß-arrestin recruitment to the MOR-DOR heteromer is disrupted by MOR or DOR ligands. These data suggest that MOR-DOR receptor heteromers adopt conformations favorable to arrestin recruitment. However, destabilization of this receptor conformation by ligand occupancy of either protomer in the MOR-DOR heteromer leads to a switch from arrestin-dependent to arrestin-independent signaling.

The kinetics of agonist-stimulated ERK activation also differs between the MOR-DOR heteromer and the individual receptors. In cells expressing MOR alone, the kinetics of DAMGO-mediated ERK activation is fairly rapid, with peak activity within 3–5 min of agonist administration. However, in cells expressing MOR-DOR heteromers, DAMGO-mediated ERK activation occurs in a sustained manner with peak activity 10–20 min following agonist administration. Similar differences in kinetics of ERK activation were found to occur with DOR agonists at DOR *versus* the MOR-DOR heteromer [60]. Understanding the coupling of ß-arrestin to opioid receptors is important given that in β-arrestin-2 knock out (KO) mice, morphine-mediated analgesia is enhanced and development of tolerance is decreased [61]. MOR-DOR heteromer receptors also appear to have altered G protein coupling preferences in comparison to MOR or DOR monomers/homomers. Agonist activation of MOR or DOR homomers/monomers typically leads to activation of pertussis toxin sensitive G_i/o_ proteins. However, in given cell types, activation of MOR-DOR heteromer receptors have been shown to preferentially activate the pertussis toxin insensitive G protein, Gz [50,62,63].

Changes in functional responses also occur upon MOR-DOR heteromerization. In an elegant study by Lakshmi Devi’s group, a potential role for MOR-DOR heteromer receptors in chronic pain has been identified [53]. Using a subtractive immunization strategy, monoclonal antibodies were generated that selectively recognize MOR-DOR heteromers but not individual MOR or DOR. The antibody was able to block heteromer-mediated ligand binding and signaling, but not binding and signaling at MOR or DOR expressed singly. Interestingly after chronic, but not acute, morphine treatment immunocytochemical staining with the heteromer-selective antibody was increased in key areas of the CNS (RVM) as well as in the periphery (DRG), suggesting that chronic morphine increases the number of MOR-DOR heteromers in areas that are involved in pain processing. Given that MOR-DOR heteromer receptors constitutively recruit and signal via arrestins, these data suggest that increased expression of heteromers may contribute to the development of tolerance under prolonged opioid (e.g., morphine) treatment conditions. Antibodies targeting GPCR heteromers will be a very useful tool to investigate endogenous heteromer involvement in effects of opioid analgesics.

In addition to antibodies, ligands have been developed which appear to selectively target MOR-DOR heteromer receptors. Portoghese and colleagues have developed a series of bivalent ligands called MDANs ((M)-delta-opioid receptor (D)-agonist (A)-antagonists (N) which contain a MOR agonist (oxymorphone) connected, through varying spacer lengths (16–25 atoms), to a DOR antagonist (naltrindole) [64]. These bivalent ligands have higher affinity for MOR-DOR receptor heteromers than monomer/homomers and are more potent than morphine for producing antinociception in the tail flick assay. Interestingly, neither tolerance nor dependence developed for ligands with spacers-19 and -21 in length whereas tolerance/dependence did develop in response to ligands with shorter spacer lengths [65]. Importantly, the effects on antinociception by these bivalent ligands are consistent with findings (discussed above) that ligand occupancy of both protomers in the MOR-DOR heteromer leads to a switch in signaling mechanisms such that antinociception is enhanced, but the development of tolerance, dependence and also perhaps rewarding behavior is decreased. In summary, the distinct signaling properties of MOR-DOR heteromers suggest that these receptor heteromers may be important therapeutic targets for treatment of chronic pain with a reduction in both tolerance and dependence liabilities.

### 2.3. DOR-KOR Heteromers

The first direct demonstration of opioid receptor heteromer formation was between DOR and KOR *in vitro* [66]. *In vivo*, the DOR-KOR heteromer is expressed in spinal cord [67,68] and trigeminal ganglia (TG) sensory neurons [69]. For DOR-KOR receptor heteromers, simply the presence of one receptor can alter the affinity and/or efficacy of a ligand at the second receptor [66,67]. Further, the ligand-receptor conformation of one protomer in the DOR-KOR heteromeric pair can influence the affinity and/or efficacy of a ligand at the other protomer [68,69,70,71]. In addition, functional responses can be either enhanced or reduced depending upon the DOR-KOR ligand pairs. For example, in a recent study by Berg *et al*. (2011) the selective KOR antagonist, nor-BNI, differentially altered the potency and/or efficacy of DOR agonists, depending upon the specific DOR agonist used [69].

Several ligands have been developed which recognize DOR-KOR heteromer receptors. Although originally developed as a KOR selective ligand, 6'-guanidinonaltrindole (6'-GNTI), has been reported to preferentially activate DOR-KOR heteromers [67,72]. In rats, 6'-GNTI produces analgesia only when administered into the spinal cord and not into brain [67]. Further, the antinociceptive potency of 6'-GNTI is greatly reduced in DOR KO mice [68]. In addition to activity in the spinal cord, 6'-GNTI was also found to produce antinociceptive responses acting in the periphery. In the rat hindpaw model of thermal allodynia, 6'-GNTI produced a more profound reduction in PGE_2_-mediated-allodynia than that seen with DOR agonists [73] or KOR agonists [74]. Further, the analgesic response to 6'-GNTI is blocked by both selective DOR and KOR antagonists, suggesting preferential targeting of DOR-KOR heteromer receptors *in vivo* [69]. Taken together, these data are consistent with the idea that DOR-KOR heteromer receptors may be tissue-specific as well as able to be activated selectively.

A bivalent antagonist as well as a bivalent agonist have also been developed which preferentially interacts with DOR-KOR heteromer receptors [68,71]. The bivalent antagonist, KDN-21, which has antagonist properties at both DOR and KOR, displays the highest binding affinity by an order of magnitude in membranes prepared from cells co-expressing both DOR and KOR [71]. The bivalent ligand, KDAN-18, which has KOR agonist activity but antagonist properties at DOR, also displays higher affinity, by approximately 100-fold, in membranes from cells co-expressing DOR and KOR receptors [64].

In addition to selective ligands, using a subtractive immunization strategy, antibodies which recognize selectively DOR-KOR heteromers have also been generated. These monoclonal antibodies recognize an epitope in cells co-expressing DOR-KOR receptors, but not other heteromer receptors (e.g., MOR-DOR), or either DOR or KOR alone [69]. Interestingly, *in vivo*, this antibody, which had no effect on its own, was found to potentiate the antinociceptive effects of the DOR agonist, DPDPE, in a rat model of thermal allodynia [69]. In the presence of this antibody, a sub-threshold (*i.e*., ineffective) dose of DPDPE became capable of not only inhibiting thermal allodynia, but producing close to the maximal possible antinociceptive response in this system.

### 2.4. MOR-KOR Heteromers

There are several reports that MOR can form heteromers with KOR, and that MOR-KOR receptor heteromers can modulate pain, particularly with drug administration into the spinal cord [75,76]. Heteromer receptor formation between MOR and KOR was demonstrated directly, in rat spinal cord membranes, using receptor subtype selective antibodies and co-immunoprecipitation techniques [75].

A selective ligand *N*-naphthoyl-à-naltrexamine (NNTA), which preferentially targets MOR-KOR receptor heteromers has been developed by Portoghese’s group [76]. NNTA was found to increase both intracellular calcium release and GTPγS binding with 1,000-fold enhanced potency in cells expressing MOR-KOR receptor heteromers *versus* cells expressing KOR alone. Interestingly, NNTA was found to be an antagonist in cells expressing MOR alone. NNTA also produces potent antinociception in the mouse tail flick assay [76]. The NNTA-mediated antinociception was more than 100-fold greater when the ligand was administered intrathecally in comparison to intracerebroventricular administration. These data suggest that a ligand such as NNTA may be particularly effective for spinal antinociception.

MOR-KOR heteromer receptors may provide an important analgesic target in women [77]. It is known that women are more likely than men to experience more severe levels of pain, with longer duration, as well as more recurrent pain [78]. Further, it has been suggested that females may respond better than males to analgesics targeting KOR [79,80,81]. Interestingly, spinal morphine antinociception in female, but not male, rats requires the simultaneous activation of spinal MOR and KOR receptors [82]. Studies suggest that the mechanism for enhanced morphine responses in female rats is formation of MOR-KOR receptor heteromers [75]. Further, MOR-KOR receptor heteromers appear to be regulated by female sex hormones since they are more prevalent during proestrous *versus* diestrous [75]. In addition, estrogen may be necessary for MOR-KOR heteromer formation in spinal cord, because blockade of membrane estrogen receptors reduced the abundance of MOR-KOR receptor heteromers detected as well as abolished KOR-mediated enhancement of spinal morphine antinociception [77].

### 2.5. MOR-Alpha Adrenergic_2A_ Receptor Heteromers

The analgesic profile of α_2_ adrenergic receptor agonists has been well established [83]. There are three subtypes of α_2_ adrenergic receptors, -2A, -2B and -2C. Analgesic responses to α_2_ agonists, particularly in the spinal cord, are mediated primarily by α_2A_ receptors [84,85]. Results of early pharmacological studies suggest that significant functional interactions occur between MOR and α_2A_receptors occur in spinal cord [86]. For example, intrathecal injection of sub-threshold doses of morphine were found to enhance the effects of clonidine and *vice versa* [87]. The combination of a subcutaneous morphine injection along with intrathecal clonidine administration produced significant antinociceptive effects, whereas either drug administered alone had no effect [88]. Further, MOR-mediated analgesia is also enhanced by the presence of α2 receptors suggesting that effective MOR-mediated spinal analgesia is dependent upon the presence of α_2A_ receptors [84].

Heteromerization between MOR and α_2A_ receptors has been demonstrated in transfected cells as well as in primary neuronal cell cultures [89,90,91]. *In vitro*, signaling is increased in response to morphine in cells co-expressing receptors in comparison with cells expressing MOR alone [89], which suggests that heteromerization alone may lead to enhanced antinociception in response to morphine. Interestingly, differences in MOR-α_2A_ receptor heteromer signaling profiles are observed in response to single drug administration *versus* co-administration of morphine and clonidine [89]. In membranes obtained from primary cultures of rat spinal cord neurons (which co-express both receptors), GTPγS binding was reduced in the presence of both morphine and clonidine relative to morphine alone [89]. Overall, these data are consistent with the idea that agonist occupancy of both protomers in the MOR-Aalpha_2A_ heteromer leads to a decrease in signal transduction whereas activation of one protomer in the complex leads to enhanced responsiveness.

### 2.6. DOR-Alpha Adrenergic_2A_ Receptor Heteromers

Although no direct demonstration of heteromerization between DOR and α_2A_ receptors has been reported, it is important to note that functional interactions between DOR and α_2A_ receptors have been shown. For example, co-incubation with the DOR agonist, deltorphin II along with clonidine produced a greater than additive reduction in K^+^-stimulated neuropeptide release from synaptosomes prepared from rat spinal cord [92]. Further, DOR and α_2A_ receptors co-localize in rat dorsal horn [92]. Therefore, along with MOR-α_2A _receptor heteromers, its quite possible that DOR-α_2A _receptor heteromers in spinal cord may play a significant role in pain modulation.

### 2.7. Cannabinoid-Opioid Receptor Heteromers

Activation of either subtype of cannabinoid receptor, CB1 or CB2, which are found in areas of the brain and in the periphery involved in pain processing, can have profound antinociceptive effects (for review see [93,94]). CB2 receptors have been shown to homodimerize [95]. However, there are no reports of receptor heteromers with CB2 receptors and other GPCRs known to be involved in pain modulation. In contrast, biochemical and pharmacological data have shown that CB1 receptors can form receptor heteromers with each of the three opioid receptors *in vitro* [96]. Although direct evidence for heteromers between CB1 and opioid receptors has yet to be determined *in vivo*, CB1 receptors do co-localize with MOR in the dorsal horn of the spinal cord [97,98] as well as in brain regions which play an important role in antinociception (e.g., the periaqueductal gray) [99,100].

Results from a number of pharmacological studies *in vivo* suggest that CB1 receptors may interact with MOR in a synergistic fashion to enhance antinociceptive responses. Thus, co-administration of the CB1 receptor agonist, delta-9-tetrahydrocannabinol (THC) synergized with morphine to enhance the antinociceptive response to a thermal stimulus in the tail flick assay in mice [101]. Further, co-administration of low doses of both CB1 and MOR agonists produced a synergistic enhancement of antinociception allowing for agonist dosing to be kept well below those which produce analgesic tolerance [102].

### 2.8. MOR-GRPR (Gastrin-Releasing Peptide) Receptor Heteromers

Scratching behavior in mice induced by diverse chemical and pharmacological agents has been shown to be mediated by activation of gastrin-releasing peptide receptor (GRPR) in the spinal cord [103,104]. The most common side effect of opioids, such as morphine, is the sensation of itch which is particularly prominent after spinal administration. Until recently, it was thought that the onset of itch was linked to the same mechanisms as the reduction in pain by morphine. However, a recent report showed that not only are the analgesic effects of morphine separable from the sensation of itch, but that morphine-induced itch is due to activation of heteromers between GRPR and MOR-1D, a splice variant of MOR, localized in spinal cord [77]. Direct protein-protein interactions were demonstrated to occur between MOR-1D and GRPR in HEK cells as well as in native spinal cord membranes [105]. Selective reduction of MOR-1D receptor expression by siRNA knockdown reduced morphine-induced scratching in mice whereas morphine-induced analgesia remained unaffected [105]. Further, blocking formation of MOR1D-GRPR receptor heteromers by injection of a TAT fusion peptide (TAT-MOR1Dct) into spinal cord led to reduction of morphine-induced itch but had no effect on morphine-induced analgesia. In addition, morphine-induced scratching, but not antinociception, was blocked in the presence of a GRP receptor antagonist. Taken together, these data strongly suggest that activation of MOR1D-GRPR receptor heteromers by morphine in mice mediates itch whereas activation of MOR mediates antinociception.

## 3. Ligand-Gated Channel Heteromers as Therapeutic Targets in Pain Pathway

Except for certain pore-forming peptides, all ion channels function as multimers, composed of varying numbers of subunits. Thus, ligand-gated channels can function as homomeric or heteromeric. These multimers form either tetrameric or pentameric structures. Throughout the last decade, the functions of these heteromeric channels in pain pathways were extensively studied. In this section, we will provide an overview of the functional and regulatory consequences of heteromerization for ligand-gated channels that have been implicated in the pain pathway. It should be noted here that a majority of voltage-gated channels also function as homo- or heteromers. However, their roles in the pain pathway have not been assessed in detail as of yet.

### 3.1. GABA_A_ Channels

Classical “gate control theory” of pain proposed by Melzack and Wall postulated that inhibitory dorsal horn neurons would control nociceptive signals coming from the periphery [106]. Time has passed and the nature of this inhibitory control has been attributed to γ-aminobutyric acid (GABA) and glycine channels. Subsequently, it was demonstrated that pharmacological blockade of GABA or glycine channels in the dorsal horn provokes pain in both animals and humans [107]. It has also been shown that a reduction in GABAergic and glycinergic neurotransmission occurs during inflammatory and neuropathic pain [108]. Accordingly, facilitation of inhibitory neurotransmission in the dorsal horn could form the basis for the treatment of chronic pain (*i.e*., antihyperalgesia and allodynia). As such, GABA_A_ receptors are among the most successfully exploited drug targets. Benzodiazepine-sensitive GABA_A_ receptors are expressed in pivotal structures of the pain pathway, including sensory neurons as well as the superficial layers of the dorsal horn [109]. Local administration of diazepam or midazolam is antinociceptive for animals [110] and is successfully utilized in human patients [111]. However, this approach is not without its drawbacks, as there are severe side effects associated with GABA_A_ agonists [112]. What are possible strategies in overcoming these difficulties? One feasible approach could be based on the hypothesis that subtypes of GABA_A_ receptors involved in anti-hyperalgesia differ from those GABA_A_ receptors involved in other CNS functions. Indeed, GABA_A_ is a classic example of a channel-heteromer, which cannot function as a monomer or homomer. GABA_A_ channels are heteropentameric chloride channels assembled from a repertoire of α, β, γ, δ, ε, θ, π, and ρ subunits. Benzodiazepine-sensitive GABA_A_ channels are assembled from two α (α1, α2, α3 or α5) subunits, two β subunits, and one γ2 subunit. All benzodiazepine-sensitive GABA_A_ channels contain a conserved histidine residue in the *N*-terminal domain, while the benzodiazepine-insensitive subunits (α4 and α6) carry an arginine at the same site [113]. Rudolph and Mohler used an elegant approach to attribute the different *in vivo* actions of benzodiazepines to molecularly defined GABA_A_ receptor subtypes [114]. They generated knock-in point mutations for all four benzodiazepine-sensitive GABA_A_ receptor α subunits (α1(H101R), α2(H101R), α3(H126R), and α5(H105R)). Using these mice, it was demonstrated that the spinal antihyperalgesic effects of diazepam were mediated mainly through GABA_A_ channels containing α2, but not α1, α3 and α5 subunits [115,116]. Furthermore, analysis of these mice and mice carrying mutations in β1 and β3 subunits also indicated that the GABA_A_ subtypes responsible for antihyperalgesia were different from those that mediate sedation [117]. This is an important observation, as these subtypes could provide the foundation for benzodiazepine-site ligands with a better side-effect profile. At present, the two relevant questions are: (1) Can other unwanted effects besides sedation also be avoided with subtype-selective agonists? (2) Does the concept of a benzodiazepine-mediated antihyperalgesia also work in humans? Both questions are at present difficult to answer. The second question has to await the availability of subtype-selective agonists suitable for clinical trials in humans. A subtype-specific GABA_A_ agonist L-838,417 possesses undesirable pharmacokinetics in man, while TPA023 and SL651498 appear more favorable [118]. It is, however, not yet clear whether these compounds would be suitable for human pain studies. TPA023 has low intrinsic activity [119], while SL651498 activates α1 subunits and could cause significant sedation when given at antihyperalgesic doses [120]. The question of whether other relevant side effects apart from sedation such as tolerance development, addiction, and memory impairment can be avoided with subtype-selective agonists is also not yet resolved. Nevertheless, some potentially important information was obtained from studies on the function of differing GABA_A_ heteromers. Analysis of α1 (H101R) point-mutated mice demonstrated that facilitation of GABA_A_α1 subunits underlies the amnestic effects of benzodiazepines [121]. However, hippocampus-dependent learning also involves α5 GABA_A_ receptors [122]. Physical dependence does not occur with several α1-sparing agents [123]. L-838,417 tolerance against antihyperalgesia was completely absent during a 9-day treatment period—in striking contrast to morphine, which had lost its analgesic activity after 6 days [115]. In summary, recent evidence obtained either with genetically modified mice or with subtype-selective GABA_A_ ligands indicates that the antihyperalgesic activity of GABA_A_ channels could be potentially separated from other unwanted side effects of the receptor. Novel subtype-selective GABA_A_ agonists are in development which not only lack undesired sedation but also avoid the other GABA_A_ agonist side effects such as memory impairment, physical dependence, and addiction.

### 3.2. Nicotinic Channels

It has recently been recognized that antagonists against nicotinic acetylcholine receptors (nAChRs) are not only able to generate acute analgesia, but are also capable of producing long lasting anti-hyperalgesia [124]. nAChRs are classic heteromeric-pentameric channels. However, unlike GABA_A_ channels, they can function as homomers. nAChRs are assembled from one or more α subunits (α1–α10) either alone or together with one or more non-α subunits (β-subunit, (β1–β4), γ, δ or ε) [125]. nAChR subunits may be divided into two broad classes. Alpha subunits have a defining “cysteine loop”, whereas non-alpha subunits lack this cysteine loop. Alpha subunits may be further divided into two groups: one group consists of α7, α9 and α10 subunits which are all potently blocked by α-bungarotoxin [126]. Both the α7 and the α9 subunit can assemble into a functional homopentamer, while the α10 subunit has only been functionally co-expressed with an α9 subunit [127,128]. Individual subtypes of nAChRs each have unique expression patterns as well as pharmacological and biophysical properties. This characteristic allows for the possibility that a variety of nAchR heteromers and homomers could each have distinctive physiological functions [128,129]. Therefore, subtype selective compounds may have distinct therapeutic applications with a restricted set of side effects. Originally, the generation of mice lacking one or more subunits of nAChRs has been utilized to examine the function of different nAChR subtypes. However, this approach is less effective, as one particular subtype of nicotinic channel can be involved in many types of nAChR. For example, there are α4β2, α4α5β2, α4β4 and α6α4β2β3 nAChR’s, deletion of the α4 nAChR subunit would eliminate all of these nAChR subtypes. This illustrates why compounds that can differentiate between these individual receptor subtypes are urgently needed. Compounds can be either chemically synthesized or isolated as toxins from different animals. In the case of nAChRs, nature has presented a unique opportunity. A bio-diverse lineage of the cone snail (500–700 different species) produces an exogenous family of toxins targeting subtypes of the nAChR family. These toxins comprise the α-conotoxin family, which contain >1,500 different α-conotoxins. Careful combination of molecular genetics, peptide chemistry and molecular phylogeny allowed exploration of the subtype selectivity of this large inventory of α-conotoxin sequences. During the last decade, a significant number of subtype specific ligands for individual nAChR isoforms were discovered. There are already detailed and comprehensive reviews on this subject [130]. In this review, we will focus on subtype-specific α-conotoxins and other AChRs antagonists which are involved in the pain pathway. Chronic injury leads to significant over-expression of key subunits including the α4, 5 and 7 subunits. This has supported the therapeutic potential of AChRs in pain control [131]. Thus, nicotinic channel agonists which are selective for the α4, 5 and 7 subunits (epibatidine, ABT-594), particularly compared to α4β2, have demonstrated antinociception in several models of pain including neuropathic pain. Several sites of action have been proposed including supraspinal sites via descending pathways and actions on sensory neurons. Earlier clinical evaluation of ABT-594 revealed inadequate tolerability, but more recent compounds such as TC6499 (GSK/Targacept) and ABT-894 (Abbott) are progressing forward for their use in diabetic neuropathy pain, and are currently in clinical phases 1 and 2, respectively [132]. α-Conotoxin RgIA is the most selective α9α10 antagonist yet reported [133]. One additional peptide that blocks α9α10 nAChRs is α-conotoxin Vc1.1 (or ACV1) [134]. The subcutaneous or intramuscular administration of α-Conotoxin RgIA and Vc1.1 acutely alleviates pain resulting from traumatic, inflammatory, or metabolic neuronal injury [135]. Thus subcutaneous administration of Vc1.1 in rats dose-dependently reverses chronic constriction injury-induced mechanical allodynia by approximately 54–80%, with higher concentrations having an extended effect lasting up to 24 hours post-administration [135]. α9α10 nAChR antagonists also show anti-hyperalgesia in the partial sciatic nerve ligation model and diabetic neuropathy [135]. Vc1.1 is an effective analgesic against pain resulting from a purely inflammatory insult generated by Complete Freund’s Adjuvant (CFA). Recent reports suggest that the anti-hyperalgesic effects of α-conotoxins Vc1.1 and RgIA could be mediated via activation of GABA_B_ receptors in DRG [136,137]. Yet other reports have shown that these toxins are not able to directly activate GABA_B_ receptors, as they have no homology to nAChRs [138]. But what are the possible off-target effects for these toxins? α-conotoxins, as charged peptides, are unlikely to cross the blood-brain-barrier in significant quantities and intrathecal administration of α-conotoxin RgIA is not analgesic in spinal nerve ligated (SNL) rats [138]. Both α9 and α10 nAChR subunits are expressed in a wide range of peripheral tissues such as sensory ganglia, heart, skin, nasal epithelium, immune cells and so on [138]. Precise off target actions for α9α10 nAChR-specific drugs are unknown. However, a role for α9α10 nAChR in anti-hyperalgesia was suggested [135]. In summary, α-conotoxins Vc1.1 and RgIA as α9α10 nAChR-specific antagonists have unique pharmacological actions and analgesic effects that may serve as models for the development of additional nicotinic channel subtype-specific drugs for pain research and management.

### 3.3. Glutamate-Gated Channels

The excitatory transmitter glutamate plays an important role in the initiation and maintenance of chronic pain conditions. Glutamate acts through ionotropic (iGluRs) as well as metabotropic receptors. Glutamate-gated channels can be classified into three groups: *N*-methyl-D-aspartate (NMDA) channels, alpha-amino-3-hydroxy-5-methylisoxazole-4-proprionate (AMPA) channels and kainate channels [139,140]. Injections of NMDA, AMPA, and kainate produce a pro-nociceptive response, whereas the administration of iGluR antagonists attenuates pain [141,142]. Molecular cloning has identified that GluR1-4 (or GluRA-D) make up the AMPA receptors, GluR5-7 and KA1-2 comprise the kainate receptors, and NR1 together with NR2A-D form the NMDA receptors [143]. Expression studies in various heterologous systems demonstrated that iGluR operate as tetramer homomeric or heteromeric complexes [144], although pentamer complexes were also suggested [145]. The AMPA and kainate receptors can form homomeric receptors, while the NMDA receptors are obligate heteromeric receptors composed of homologous NR1, NR2, and/or NR3 subunits. A large fraction of the neuronal NMDA channels are composed of glycine binding NR1 and glutamate binding NR2 subunits [146]. It was suggested that NMDA channels have a dimer arrangement, with each dimer having one glycine binding subunit and one glutamate binding subunit [147]. The NMDA channel heteromers have distinctive biophysical, pharmacological and physiological properties [146]. Thus, replacement or supplementation of NR2B by NR2A during development has been implicated in the acceleration of NMDA-EPSC decay, a phenomenon often linked with the ability of neuronal circuits to exhibit experience-dependent synaptic plasticity [148]. This type of change was described in many areas of the brain, including visual cortical cells, cerebellar granule cells, hippocampal CA1 pyramidal cells and the anterior neostriatum. All of these cells display a change in the NMDA-EPSC kinetics consistent with a decreasing contribution of NR2B subunits and an increasing synaptic involvement of NR2A, which is responsible for faster NMDA-EPSC. These changes also lead to the significant alteration of Ca^2+^ signaling that could affect the synaptic conductance. How could these NMDA heteromer specific drugs be employed in pain management? NMDA antagonists show robust attenuation of pain but come with a number of side-effects (sedation, confusion, and decrease motor coordination) and thus appear to have insufficient therapeutic margin. In an attempt to avoid these side-effects, specific blockers of NMDA receptor subtypes (NR1 and NR2) are being developed. Consequently, antagonists against glycine sites could modulate the NMDA channel during sustained receptor stimulation found to occur during chronic pain. Accordingly, selective NR1-glycine site antagonists reduce pain with reduced side-effects [149]. Unfortunately, clinical experience with this compound, GV196771 did not show efficacy against clinical pain [150]. The NR2B receptor has a specific distribution in sensory pathways and as mentioned slows NMDA current kinetics [151]. Blockade of this receptor has also been reported to produce antinociception (ifenprodil, traxoprodil, CP-101606) with reduced side-effects [152]. To date, traxoprodil has advanced into phase 1 safety and efficacy studies for acute ischemic stroke and there is as of yet little information about possible developments in its use in treating pain. However, other NR2B projects including RGH 896 and EV101 have reached phase 2 and were evaluated for cognition and neuropathic pain [151]. The roles of AMPA and kainate channel heteromers in the pain pathway have yet to be studied. However several antagonists against AMPA and kainate channels were implicated in control of nociceptive processing during chronic pain conditions [153,154].

### 3.4. P2X Channels

P2X channels contain seven members and belong to the non-selective Ca^2+^-permeable cation channel family [155]. The P2X channel typically functions as a homomer. The number of subunits of a P2X channel has been proposed to be three [156] or four [157]. However, P2X can form at least four heteromeric complexes [158,159]. Homomeric and heteromeric P2X channels display different electrophysiological and pharmacological properties [160]. P2X4 and P2X7 were implicated in pain signaling in the dorsal horn [161,162], where activated microglia after nerve injury increased the expression of P2X4 and P2X7. The P2X4 and P2X7 receptors are activated by ATP which is presumably released from primary sensory neurons. P2X receptor activation causes a rise in the intracellular calcium, along with the release of diffusible factors, such as BDNF and other pro-inflammatory cytokines and chemokines. This affects excitatory and inhibitory neurons in the spinal cord, increasing the net hyper excitability of the dorsal horn in the pain network and may be responsible for neuropathic pain. So far, P2X4 or P2X7 heteromerization between other P2X channels and their respective role in the activation of microglia has yet to be reported. Furthermore, roles in pain and other physiological process of reported P2X heteromers are not yet clear.

### 3.5. TRP Channels

TRP channels are comprised of multiple subfamilies, including TRPC, TRPM, TRPV, TRPP and TRPN [163]. TRP channels have unique characteristics in terms of cell-type expression patterns [164], modes of activation [165], pharmacological profile and biophysical properties including both voltage-dependency and ion permeability [163]. It is now accepted that TRP channels function as homo-tetramers [7,24,166,167]. Accumulated evidence also demonstrates that TRP channels of the same subfamily are able to form heterotetrameric channel complexes [7]. The formation of various TRP channel complexes of TRPC [168,169,170], TRPV [23,171,172,173], TRPM [174], or TRPP [7] channels has been demonstrated. Some of these heteromers could be formed in neurons involved in nociceptive signal transmission [7,10,172].

In humans and non-human primates, TRPV1 and TRPV3 are co-expressed in sensory neurons of DRG [172,175]. Like many known heteromers, the TRPV1/TRPV3 heteromer exhibits unique biophysical and functional properties [24,173,176]. Homomeric TRPV1-V4 channels assemble into a tetramer by interacting via their *C*-terminal domains [167]. However, it appears that this region does not play a critical role in heteromerization between TRPV1 and TRPV3 [173]. TRPV1/TRPV3 heteromeric channels are different from TRPV1 and TRPV3 homomeric channels in regards to sensitivities and dynamic range to thermal and chemical stimuli, but are similar in regards to changes in transmembrane voltage [173]. These observations indicate that they serve as distinct cellular sensors to thermal and chemical stimuli, and possibly mediate different sensory responses to a variety of stimulants. Like other heteromeric channels, temperature-sensitive TRPV channels are known to be allosteric proteins [177]. Cooperative gating among subunits shifts the sensitivity and dynamic range exhibited by subunits in homomeric channels. These properties must be carefully considered when examining the response of native neurons to heat and agonists.

Recently, formation of a heteromeric complex in sensory neurons between TRP channels belonging to different subfamilies (TRPA1 and TRPV1) has been revealed [10,22]. The heteromerization and subunit composition can influence the biophysical and regulatory properties of the resulting channel complex [10], revealing unique pharmacological properties [178,179]. This includes altering the stability of receptors on the plasma membrane by governing the trafficking properties of the receptors [180,181,182]. Desensitization of receptors can also be influenced by complex formation [25,183,184,185]. The sensitization modes of receptors by different cell stimulants (such as inflammatory mediators) could be regulated in a unique way within these heteromeric complexes. Based upon these findings, it is possible that a functional interaction between TRPA1 and TRPV1 channels may occur via a variety of pathways within a heteromeric complex [185,186,187,188,189].

Regulation of TRP channels within homomers by a variety of extracellular and intracellular soluble factors has been investigated in detail. The physiological roles of a majority of TRP channels are well described and assessed. However, the regulation and especially the physiological functions of heteromeric TRP complexes are less understood compared to other ligand gated channels discussed in this review. Most notably the TRPA1/TRPV1 heteromeric complex formed in the plasma membranes of sensory neurons and found in pain pathways [22]. Here we will focus on the regulation and possible functions of this heteromeric complex. A stoichiometry of the TRPA1/TRPV1 complex is unknown, and the percentage of sensory neurons in trigeminal (TG) and dorsal root ganglia (DRG) expressing functional TRPA1/TRPV1 heteromers is also unclear. However, it is presumed that all TRPA1 and TRPV1 co-expressing sensory neurons contain functional TRPA1/TRPV1 heteromers. It has been shown that the percentage of TRPA1 and TRPV1 co-expressing neurons is up-regulated by inflammation and NGF [190]. At physiological and un-inflamed conditions (Vh = −60 mV; 2 mM extracellular Ca^2+^) the biophysical properties of TRPA1 is regulated by the presence of TRPV1 channels [10,22,27]. TRPV1 also controls the density of functional TRPA1 channels on the plasma membrane [25]. It is still not clear whether TRPA1 density is controlled by the TRPV1 channel on a transcriptional, translational and/or post-translational level. TRPV1 can also modulate the pharmacological properties of the TRPA1 channel. Thus, the TRPA1 agonists AM1241 (synthetic cannabinoid activating CB2 receptor) and AM630 (synthetic antagonist for CB2) are more potent in the presence of TRPV1 [178,179].

There are several possible mechanisms underlying the functional interaction between TRPA1 and TRPV1 channels. First, capsaicin and mustard oil pretreatments result in pharmacological [191,192] and functional [193,194] cross-desensitization between TRPA1 and TRPV1. Mustard oil desensitizes TRPV1 via a Ca^2+^-dependent mechanism involving the Ca^2+^-dependent phosphatase, calcineurin [25,192]. Mustard oil triggers Ca^2+^-influx into sensory neurons and activates calcineurin, which then dephosphorylates and desensitizes the TRPV1 channel. Desensitization of TRPA1 by capsaicin is also Ca^2+^-dependent [192], but this process employs Ca^2+^-evoked depletion of PIP_2_ [25]. Additionally, in TRPA1-TRPV1 containing sensory neurons, it appears that TRPV1 also governs desensitization of TRPA1 by inhibiting the internalization process for TRPA1 [25]. However, it is still not clear whether heteromerization between TRPA1 and TRPV1 can directly contribute to desensitization between these channels. Nevertheless, such cross-desensitization between TRPA1 and TRPV1, which plays a critical role in inflammatory hyperalgesia, could have therapeutic implications. Thus, inhibition of sensory neurons via certain cannabinoids (including WIN55, 212-2, AM1241) activating TRPA1 can lead to functional desensitization of the pain processes in sensory neurons mediated by TRPV1 [178,185].

Functional interactions between TRPA1 and TRPV1 channels may take place within a heteromeric complex. There are three possible pathways that may underlie this interaction. (1) Bradykinin (BK) can indirectly activate TRPA1, possibly via generation of diacylglycerol (DAG) accumulation [195]. TRPV1, as a component of the complex, may act as a modulator that is responsible for sensitization of TRPA1-mediated BK responses; (2) The pharmacological desensitization of TRPA1-mediated responses in sensory neurons lacking TRPV1 is more pronounced [25]. Therefore, it could be suggested that the absence of TRPV1 in sensory neurons may lead to a faster desensitization of BK responses in sensory neurons, and this, in turn, may suppress the development of inflammatory hyperalgesia. Indeed it was reported that an increased rate of functional desensitization of BK-induced action potentials was found in TRPV1 KO mice peripheral sensory fibers [196]; (3) Finally, TRPA1-mediated BK responses in sensory neurons could be modulated by a combination of TRPV1 and [Ca^2+^]_i_. Thus, [Ca^2+^]_i_ may influence ligand affinity and/or signaling or other properties of a TRPA1-TRPV1 complex. In summary, to understand the mechanisms underlying functional interaction between the TRPA1 and TRPV1 channels, TRPA1 and TRPV1 *in vitro* and *in vivo* characteristics need to be studied in the presence and absence of TRPV1 and TRPA1 channels, respectively.

## 4. Approaches To Design Inhibitors for Heteromer Receptors and Channels

Currently, there are multiple options for the screening of drugs which are dependent on the nature of the therapeutic target and on the strategy of the laboratory or company involved in drug discovery. The existence of receptor dimers, especially of receptor and channel heteromers, gives rise to concerns about the use of monomer-based strategies. At the same time, the existence of receptor heteromers opens exciting new possibilities for drug development. Traditionally, pharmaceutical companies have utilized single receptor screening methods to identify candidate drugs. Although this approach has been successful, many of the clinical effects imparted by the drugs discovered utilizing this method have not always been predicted by the results from screening against a single receptor subtype. Further, recent analysis of the mechanism of action of some clinically used drugs has demonstrated that these drugs may be more heteromer specific and thus offer unique advantages. The atypical antipsychotic, clozapine, is an example for how targeting a GPCR heteromer may produce therapeutically effective results. Originally identified as a mixed dopamine and serotonin receptor antagonist, recently clozapine has been shown to cause dissociation of dopamine D1-D2 receptor heteromers [197]. This unique mechanism of action of clozapine offers several advantages over a typical antipsychotic such as haloperidol and is now the preferred therapy in treatment resistant patients [197]. Similar success has been seen with the smoking cessation drug, varenicline, which targets the α4β2 multimer of nicotinic acetylcholine receptors [198]. Since certain pathological states may lead to increased heteromerization, a strategy for identifying molecules that specifically target heteromers may unveil drugs that result in lesser side effects. Indeed, several new companies including Cara Therapeutics and Dimerex hope to benefit from such an approach.

Early investigations in drug discovery should first identify if a drug target of interest consists of a receptor/channel monomer, homodimer or heterodimer. If receptor monomers or homodimers are the assumed target, and screening is based on second-messenger readouts, then no modifications in the current approach are required. Yet, if radioligand binding is used in the screening of drugs targeting receptor homodimers, then dimer-based binding data will differ from monomer-based models. Finally, drug discovery must consider that drug targets may in fact be receptor heteromers, with unique properties that differ from their constitutive homomers.

As mentioned above, receptor heteromers often display unique pharmacological properties in comparison with their individual receptors [5,6,35,199]. Drugs that are selective for receptor heteromers typically have higher affinity. For example, the affinity of caffeine for the A_2A_ receptor is one order of magnitude lower for the receptor monomer/homomer than for the A_2A_-A_1_ receptor heteromer [200]. Compound screening should be performed in parallel with cells expressing both monomers/homomers as well as heteromers. It is possible that compounds discarded as having low affinity for a given receptor may actually exhibit a higher affinity for receptor heteromers, revealing a new class of receptor heteromer-selective drugs.

Receptor heterodimers may be exploited to increase therapeutic efficacy in a variety ways. As described above, small molecules that specifically bind and activate or inhibit a heteromer could be developed. Secondly, monoclonal antibodies that inhibit heteromer formation could be designed. The success of Trastuzumab and Pertuzumab which demonstrate that monoclonal antibodies can inhibit dimerization of the HER2/neu receptor in breast cancer patients suggests that such an approach is viable [201]. Moreover, monoclonal antibodies developed using a subtractive immunization strategy have been shown to recognize selectively a given receptor heteromer and alter (either enhance or inhibit) ligand-mediated effects [53,69]. Third, gene therapy may be employed as a useful strategy. For example, novel therapeutic approaches such as siRNAs can be utilized to target heteromers where the siRNA selectively disrupts the multimerization domain of a given receptor or channel, offering a significant advantage over nonspecific silencing of individual receptors/channels.

Fourth and finally, a heteromer specific blocker (or inhibitor) may be developed using a non-conventional strategy utilizing RNA inhibitors or aptamers from an RNA library [202,203]. This approach relies on RT-PCR to amplify desired RNA molecules by exponential enrichment of their sequences over background, through multiple iteration cycles, against a specific target receptor or channel. This approach provides a great advantage because it does not require a prior knowledge of the structure of the receptor/channel target. Furthermore, aptamers by themselves do not cross the plasma membrane, and could target the extracellular domains of receptors channels [204], or utilizing lipid micelles, could be used for targeting the active intracellular domains of channels such as TRPV1 [205].

## 5. Conclusions

Receptors and channels can form heteromeric complexes on the plasma membrane. We are just now beginning to understand the characteristics of different receptor and channel heteromerization in cells involved in nociceptive processing. There are functional, regulatory and pharmacological outcomes of heteromerization that are beginning to be acknowledged for a set of receptors and channels engaged in pain. Thus, these heteromeric receptors and channels display unique and specific pharmacological, biophysical, regulatory and functional characteristics. Therefore, heteromer complexes may be considered to be novel and distinct entities with the potential for playing unique physiological roles during normal and pathological conditions. Heteromers provide valid and distinct targets for agonists/antagonists development. This concept is currently overlooked by pharmaceutical companies, which concentrate on a single or homomeric receptor/channel by using expression systems in which heteromers cannot occur. In summary this area of research is well worth the effort and investment, as targeting receptor/channel heteromers may provide a completely new therapeutic strategy for the treatment of different pain conditions.
